# Incommensurate structure of AlPO_4_-5 and its stability

**DOI:** 10.1107/S205252062600449X

**Published:** 2026-05-26

**Authors:** Kazuki Komatsu, Takuji Ikeda, Tetsuya Kodaira

**Affiliations:** a Geochemical Research Center, Graduate School of Science The University of Tokyo Hongo 7-3-1, Bunkyo-ku Tokyo Tokyo 113-0033 Japan; b Research Institute for Chemical Process Technology National Institute of Advanced Industrial and Science and Technology 4-2-1 Nigatake, Miyagino-ku Sendai Miyagi 983-8551 Japan; c Research Institute for Chemical Process Technology National Institute of Advanced Industrial and Science and Technology 1-1-1 Higashi Tsukuba Ibaraki 305-8565 Japan; Academy of Sciences of the Czech Republic, Czechia

**Keywords:** Incommensurate structure, AlPO_4_-5, single-crystal X-ray diffraction, AlPO4-5

## Abstract

The incommensurate structure of AlPO_4_-5 and its stability have been investigated in detail. Under ambient conditions, single-crystal X-ray diffraction measurements reveal weak satellite reflections, which become significantly enhanced upon exposure to vacuum. Temperature-dependent X-ray diffraction and Raman scattering measurements of the O–H stretching region show that the appearance of satellite reflections is due to the desorption of water molecules under vacuum, and the satellite reflections disappear above 335 (5) K, whereas their intensity increases again upon cooling to room temperature. To elucidate the origin of the modulation, single-crystal X-ray diffraction experiments were performed on a dehydrated sample. Diffraction data including satellite reflections up to second order were successfully collected, with a modulation vector of **q** = 0.36710 (5)c*. Analysis of systematic absences up to second-order satellites indicate that the (3+1)D superspace group is *P*6(00γ)*h*. On the basis of these data, the first-order positional modulation amplitudes were refined. The results indicate that the modulation originates from correlated rotational displacements of AlO_4_ and PO_4_ tetrahedra. This behaviour suggests that phonon-like motions, as proposed in previous studies, persist over a long modulation period and give rise to the observed satellite reflections. These findings highlight a fundamental connection between the intrinsically spatial description of atomic positional modulations in the incommensurate structure and the underlying temporal dynamics.

## Abstract 2

The incommensurately modulated structure of AlPO_4_-5 was elucidated by single-crystal X-ray diffraction, revealing correlated rotational displacements of AlO_4_ and PO_4_ tetrahedra.

## 1. Introduction

Aluminophosphate molecular sieves have been widely studied since their discovery in the 1980s (Wilson *et al.*, 1983 ▸) owing to their unique adsorption properties and catalytic applications by the substitution of Al and P atoms with the heteroatoms of Si, Co, and so on (Kang *et al.*, 2008 ▸; Li *et al.*, 2025 ▸) . Numerous studies have focused on the physical and chemical properties of one of the aluminophosphate family, AlPO_4_-5, such as water adsorption (Li *et al.*, 2023 ▸), membrane filtration (Khamis *et al.*, 2023 ▸), and laser activation (Zhu *et al.*, 2024 ▸). However, despite decades of study, its detailed crystal structure remains incompletely understood.

**Figure 1:**
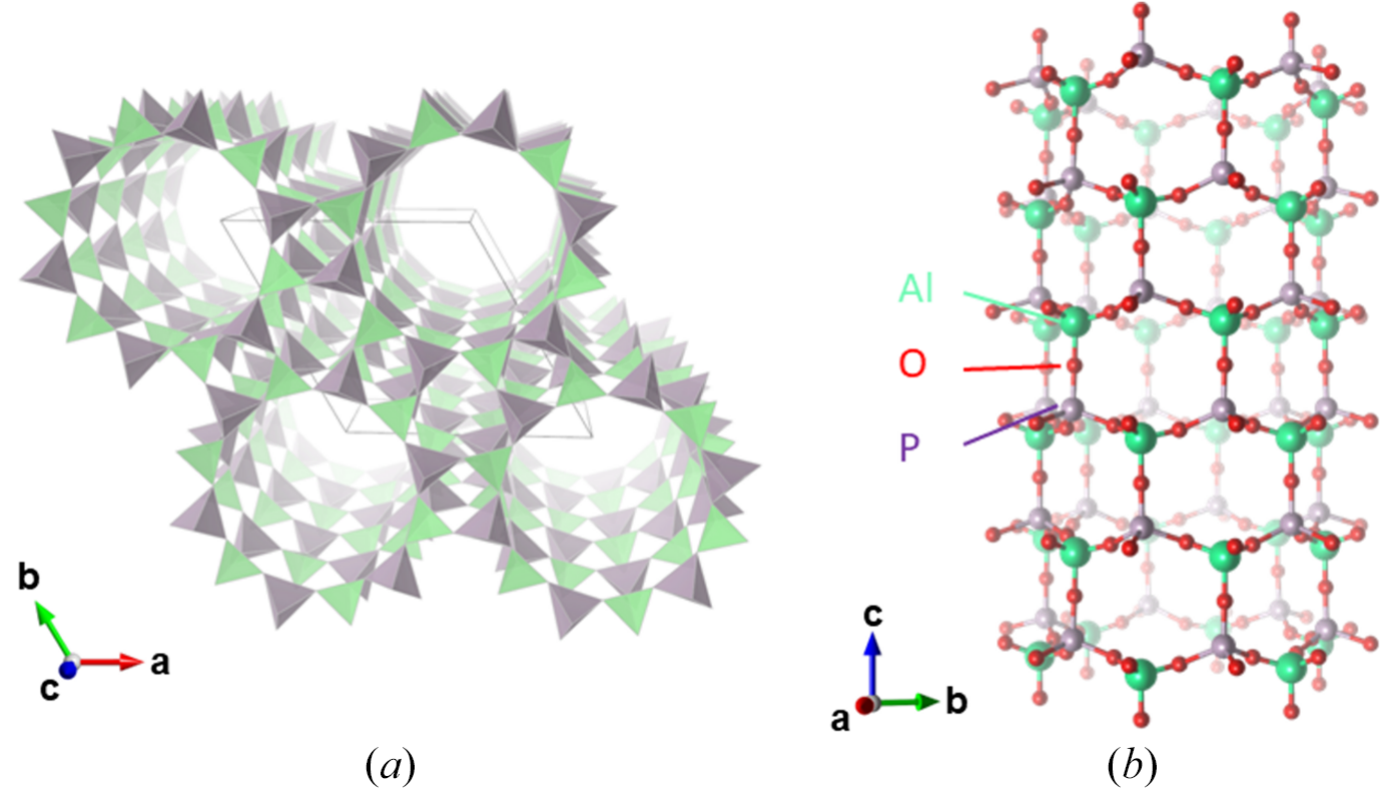
(*a*) Polyhedral representation of the crystal structure of AlPO_4_-5. Green and purple tetrahedra depict AlO_4_ and PO_4_ tetrahedra, respectively. Oxygen atoms are not shown. (*b*) Ball-and-stick representation of the 12-membered ring in the crystal structure of AlPO_4_-5.

Earlier single-crystal X-ray diffraction studies of as-synthesized AlPO_4_-5 with organic structural directing agents (OSDAs) (Bennett *et al.*, 1983 ▸, Qiu *et al.*, 1989 ▸), or time-of-flight powder neutron diffraction of the calcined material (Richardson *et al.*, 1987 ▸) have revealed its fundamental framework structure within the AFI-type zeolite family with space group *P*6*cc*. The structure is characterized by channels formed by 12-membered rings of AlO_4_ and PO_4_ tetrahedra stacked along the *c* axis, forming a one-dimensional channel of approximately 7.3 Å in diameter (Fig. 1 ▸). Additionally, owing to the charge difference between Al and P, as well as the overall symmetry, the structure exhibits polarity. This polarity has been experimentally confirmed through pyroelectricity measurements (Klap *et al.*, 1998 ▸).

A subsequent powder X-ray diffraction study including fluoride anions and tropine as an OSDA showed a reversible phase transition from hexagonal to orthorhombic space group *Ccc*2 (Ohnishi *et al.*, 1993 ▸), and further powder neutron and X-ray diffraction studies for the calcined sample also showed *Pcc*2 (Mora *et al.*, 1996 ▸, Ikeda *et al.*, 1999 ▸), a subgroup of *Ccc*2, as the most plausible space group. Subsequent computational studies of AlPO_4_-5 without OSDAs provided important suggestions that the symmetry reduction to *P*6 from *P*6*cc* resolved the unnatural linear Al–O–P angle in the *P*6*cc* structure (Henson *et al.*, 1996 ▸), and the structure with the space group *Pcc*2 is very similar to the *P*6 structure (Ruiz-Salvador *et al.*, 1996 ▸). Klap and co-authors conducted detailed structural analyses using single-crystal X-ray diffraction and proposed that the crystal structure of AlPO_4_-5 consists of three types of microdomains, each exhibiting *P*6 symmetry, and the averaged structure has the space group *P*6*cc* (Klap *et al.*, 1998 ▸).

The crystal structure of AlPO_4_-5 has also attracted attention in terms of the structure and behaviour of guest water molecules. For example, Floquet *et al.* proposed a model in which water molecules inside the 12-membered rings form a double helix (Floquet *et al.*, 2004 ▸), but this model has not been experimentally verified and may not be entirely plausible, considering the channel size. Computational studies have suggested that the guest structure varies depending on the number of water molecules present (Demontis *et al.*, 2012 ▸). Additionally, extensive research has been conducted on the relationship between the polarity of guest molecules and that of the host framework (Klap *et al.*, 1998 ▸), as well as the interaction between water dynamics and the channels (Gotoh *et al.*, 2000 ▸).

Several intriguing recent findings come from Lotti *et al.* and Fischer *et al.* (2026 ▸). Lotti *et al.* (2016 ▸) observed satellite peaks at **q** ∼ 0.37c* in X-ray diffraction patterns using a synchrotron source, indicating the presence of an incommensurate modulated structure. A recent study by Fischer *et al.* (2026 ▸) has investigated the dynamic disorder in AlPO_4_-5 using single-crystal diffuse scattering in combination with *ab initio* molecular dynamics simulations. Their work revealed the presence of correlated atomic displacements within the framework and provided important insights into the origin of the observed diffuse scattering. In particular, the study suggested that the structural fluctuations can be interpreted in terms of collective motions resembling phonon-like behaviour. Despite these advances, the detailed structural analysis of the modulation in AlPO_4_-5 has remained unexplored. Furthermore, the stability of the modulated structure with respect to temperature, pressure, and guest water molecules has not been systematically investigated.

In this study, we utilized relatively large single crystals [0.1 × 0.07 × 0.07 (mm)] to perform X-ray diffraction analysis, allowing us to investigate the previously uncharacterized modulated structure of AlPO_4_-5. Furthermore, Raman spectroscopy under high-temperature and vacuum conditions was employed to explore the relationship between the modulated structure and guest water molecules, providing new insights into the structural dynamics of this material.

## 2. Experimental

### 2.1. Sample preparation

To synthesize single crystals of AlPO_4_-5, Alumisol-10 (10 wt%, Kawaken Fine Chemicals), orthophospho­ric acid (85 wt% Sigma-Aldrich), and tri­ethyl­amine [(C_2_H_5_)_3_N, +99%, Tokyo Chemical Industry] were used as Al and P sources, and an OSDA, respectively. Deionized water was used to dilute the ingredients. The synthetic solution was obtained by mixing these ingredients in the ratios of 1.0 Al_2_O_3_: 1.2 P_2_O_5_: 4.0 (C_2_H_5_)_3_N: 550 H_2_O. We referred to previous articles for the actual procedure to mix the ingredients (Demuth *et al.*, 1995 ▸, Kodaira *et al.*, 1999 ▸). The initial solution (pH = 7.15) was adjusted to pH = 2.80 by dropwise addition of concentrated sulfuric acid (98−99%, Fujifilm Wako). This solution was sealed in a Teflon-lined autoclave and was placed in a preheated oven at 463 K for 68 h. After the hydro­thermal reaction, AlPO_4_-5 crystals were collected, washed with deionized water, filtered, and dried at 333 K. Prior to the following measurements, the crystals were calcined at 1073 K for 30 h under a dried air flow of 0.5 L min^−1^ to remove the OSDA.

### 2.2. X-ray diffraction and Raman spectroscopy at high *T*

**Figure 2:**
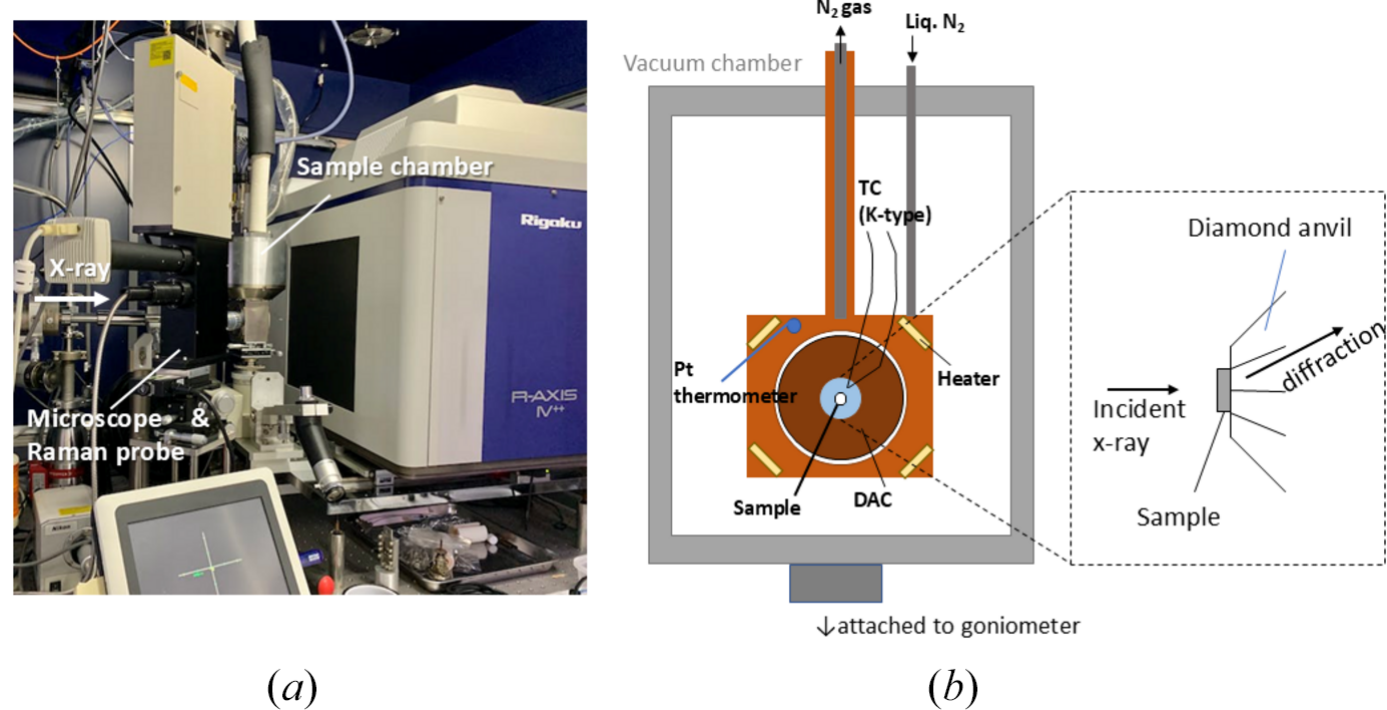
(*a*) A photograph of instruments used in this study. The sample chamber is set on the goniometer of X-ray diffractometer with a microscope and a Raman probe. (*b*) Schematic drawings for the sample chamber and the enlarged side view around the sample.

To ensure that both measurements of X-ray diffraction and Raman spectra were conducted under identical conditions on the same sample, an *ad hoc* sample chamber originally designed for low-temperature high-pressure measurements was used for ambient-pressure experiments (Fig. 2 ▸). The single-crystal sample was placed on one of the diamonds in a diamond anvil cell (DAC) made of CuBe alloy [Fig. 2 ▸(*b*)], and the DAC was mounted onto a copper frame. A liquid nitro­gen cooling line and a ceramic heater were embedded in the copper frame, and the temperature was controlled using an external temperature controller (CHINO, KP-1000), while monitoring the temperature using a platinum resistance thermo­meter embedded within the frame. The flow rate of exhausted nitro­gen gas, evaporated from the liquid nitro­gen passing through the copper frame, was controlled using a mass flow controller. The entire copper frame was housed inside an aluminium sample chamber, allowing measurements to be performed under atmospheric pressure or vacuum conditions. This chamber was equipped with two windows, one made of a polyester film (Mylar) for optical observation and Raman spectra measurements, and the other was a polyimide film (Kapton) for X-ray diffraction.

The sample temperature was monitored using a K-type thermocouple fixed near the diamond on which the sample was placed. Within the experimental temperature range (298–340 K), the temperature difference between the thermocouple and the platinum resistance thermometer in the copper frame reached a steady state within approximately 10 min after changing the set temperature. In the steady state, the temperature difference was within 0.5 K, and given the excellent thermal conductivity of the diamond, the sample temperature was estimated to be accurately monitored within approximately ±1 K.

For X-ray diffraction measurements, a rotating anode X-ray source (Mo *K*α, Rigaku, MicroMax-007) was used. The incident X-ray beam was focused onto the sample area using a confocal X-ray mirror (Rigaku, VariMax-Mo) and further collimated with a single-pinhole collimator with a hole radius of ϕ = 0.3 mm. Diffraction images were collected using an imaging plate detector (Rigaku, R-AXIS IV^++^). The sample chamber was mounted onto the goniometer of the X-ray diffractometer, and oscillation photographs were obtained by exposing the sample for 10 min while oscillating it within a φ range of −14° to −8° to observe the satellite peaks.

Raman spectroscopic measurements were also conducted on the same sample inside this chamber used for X-ray diffraction measurements. A 50 mW DPSS laser (Airix) emitting green laser light at a wavelength of 532 nm was introduced into a confocal optical system placed beside the X-ray diffractometer via an optical fibre. The backscattered light from the sample passed through a narrow-band notch filter within the optical system and was then transmitted through an optical fibre to a spectrometer (Bruker Optics 250, grating of 1200 mm^−1^), where the spectra were recorded using a high-sensitivity CCD (Andor, iDus). Raman spectra were measured in the OH stretching region at 298 K under atmospheric pressure and at 300 K, 310 K, and 320 K under vacuum (≃ 10^−1^ Pa).

### 2.3. X-ray diffraction for detailed structure analysis at room temperature

The single-crystal sample was placed in a drying oven at 373 K for 1 h in order to completely dehydrate the sample. To prevent moisture absorption, the sample was immediately coated with an adhesive upon removal from the oven. After mounting the sample on a glass fibre, it was placed on the goniometer of an X-ray diffractometer (Rigaku, Synergy Custom). The X-ray source used was a rotating anode (Mo *K*α, Rigaku, MicroMax-007), and the beam was focused onto the sample area using a confocal X-ray mirror (Rigaku, VariMax-MoHF) and collimated with a 0.3 mm collimator. Diffraction images were collected using a hybrid photon-counting X-ray detector (Rigaku, HyPix-6000HE). The obtained diffraction images were processed using *CrysAlisPro* (v. 1.171.42.70a; Rigaku Oxford Diffraction). The 3D averaged structure analysis based on the main reflections was performed with *SHELXL* (Sheldrick, 2015 ▸) implemented in *Olex2-1.5* (Dolomanov *et al.*, 2009 ▸), and the electron density distribution in the unit cell of AlPO_4_-5 was estimated using the maximum entropy method (MEM) implemented in the *Dysnomia* program (Momma *et al.*, 2013 ▸). The crystal structures with the electron density were visualized using *VESTA3* (Momma & Izumi, 2011 ▸). The analysis of the modulated structure, including satellite reflections, was performed using *JANA2020* (Petříček *et al.*, 2023 ▸). The initial model for the modulated structure was derived using the charge flipping algorithm implemented in *SUPERFLIP* (Palatinus & Chapuis, 2007 ▸).

## 3. Results and discussion

### 3.1. Stability of the incommensurate structure

During the initial screening to verify the crystallinity of the sample, we confirmed the presence of satellite reflections at approximately **q** ∼ 0.37c*, as reported by Lotti *et al.* (2016 ▸), even at ambient conditions. However, the intensity of these satellite reflections varied significantly between samples, with some exhibiting almost no observable satellites. Notably, samples that showed no satellite reflections had been exposed to air for extended periods since they were taken out of the sample bottle. This observation led us to hypothesize that moisture absorption from the atmosphere could influence the intensity of the satellite reflections.

**Figure 3:**
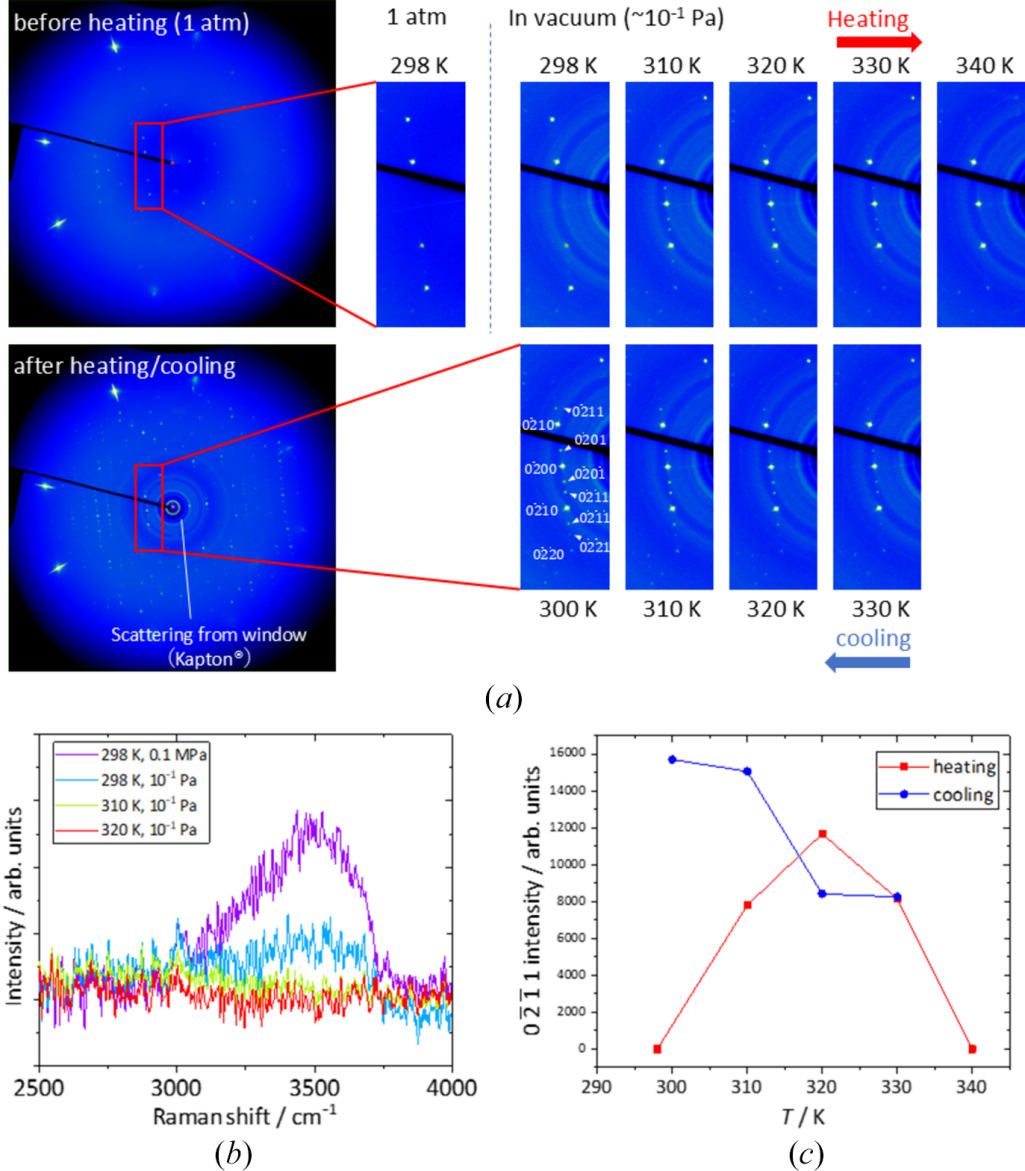
(*a*) X-ray oscillation photographs of AlPO_4_-5 during a heating–cooling cycle. The upper-left panel shows the diffraction pattern at 298 K under atmospheric pressure together with its magnified view. The subsequent enlarged images to the right (upper row) were collected during heating under vacuum, while those in the lower row correspond to cooling under the same vacuum conditions. The lower-left panel shows the diffraction pattern after the heating–cooling cycle at 300 K. The powder rings observed in the vacuum measurements originate from scattering by the Kapton window and are absent in the ambient-pressure image. (*b*) Raman spectra measured at 298 K under atmospheric pressure and at 298 K, 310 K, and 320 K under vacuum. (*c*) Intensities of a satellite peak, 0211 reflection, recorded during the heating and cooling process.

To investigate this, we placed a single-crystal sample with weak satellite reflections under vacuum. As a result, faint satellite reflections became observable after evacuation [Fig. 3 ▸(*a*)]. Raman spectroscopy further revealed that placing the sample under vacuum alone caused a significant decrease in the intensity of peaks associated with OH stretching vibrations [Fig. 3 ▸(*b*)]. Moreover, as the temperature was gradually increased in 10 K increments, the OH stretching peak became nearly undetectable at 310 K, coinciding with an increase in the intensity of the satellite reflections [Fig. 3 ▸(*c*)]. With further heating, the intensity of the satellite reflections peaked at 320 K and then began to decrease, becoming almost undetectable at 340 K. Upon cooling, the satellite reflections reappeared at 330 K, and their intensity increased progressively as the temperature decreased. During the heating and cooling process, the positions of the satellite peaks did not change significantly.

These results indicate that the satellite reflections are most intense when the water molecules are fully evacuated from the structure. Additionally, we identified an incommensurate–disordered phase transition occurring at approximately 335 (5) K. This transition temperature is slightly lower than the transition at 380 K from hexagonal to orthorhombic symmetry observed in powder X-ray diffraction studies of AlPO_4_-5 containing OSDA molecules (Ohnishi *et al.*, 1993 ▸). Furthermore, it closely matches the temperature at which the pyroelectric coefficient and spontaneous polarization begin to increase (Klap *et al.*, 1998 ▸). They also reported that the temperature at which these properties change can vary depending on the presence and type of OSDA molecules. Although modulated structures have not been reported in AlPO_4_-5 containing OSDA molecules, the atomic displacements responsible for the long-period modulation, which will be discussed in the next section, strongly suggest a connection to the symmetry reduction observed in previous studies.

### 3.2. Analysis of 3D averaged structure

**Figure 4:**
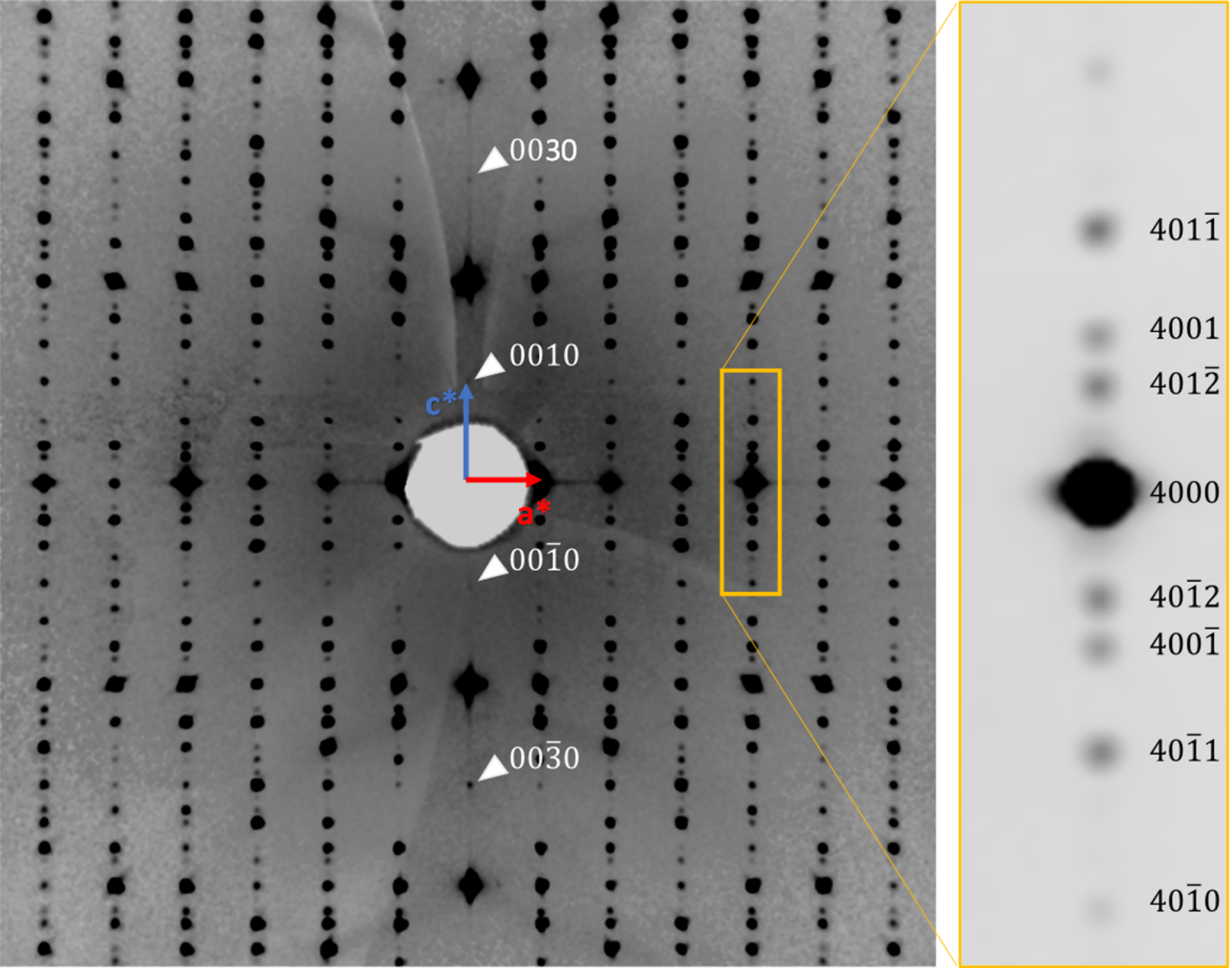
Pseudo-precession images of AlPO_4_-5 showing *h0lm* reflections. The right inset shows an enlarged reciprocal area around the 4000 reflection, which exhibits second-order satellites and reduced contrast was applied to clarify each satellite peak.

We revisited the crystal structure of AlPO_4_-5 using the observed main reflections, which represent the 3D averaged structure projected from the (3+1)D superstructure. We observed many strong *hhl* reflections (*l* = odd), which clearly violate the reflection conditions (*hhl*: *l* = even) of the previously proposed space group *P*6*cc*. We also observed weak but significant intensities for the 001 and 003 reflections (Fig. 4 ▸), indicating that no systematic extinction conditions are present. This observation suggests that the true symmetry of AlPO_4_-5 is lower than that of the space group *P*6*cc*. Assuming that the true space group is a subgroup of *P*6*cc* and considering the absence of extinction conditions, *P*6 is a plausible candidate.

**Figure 5:**
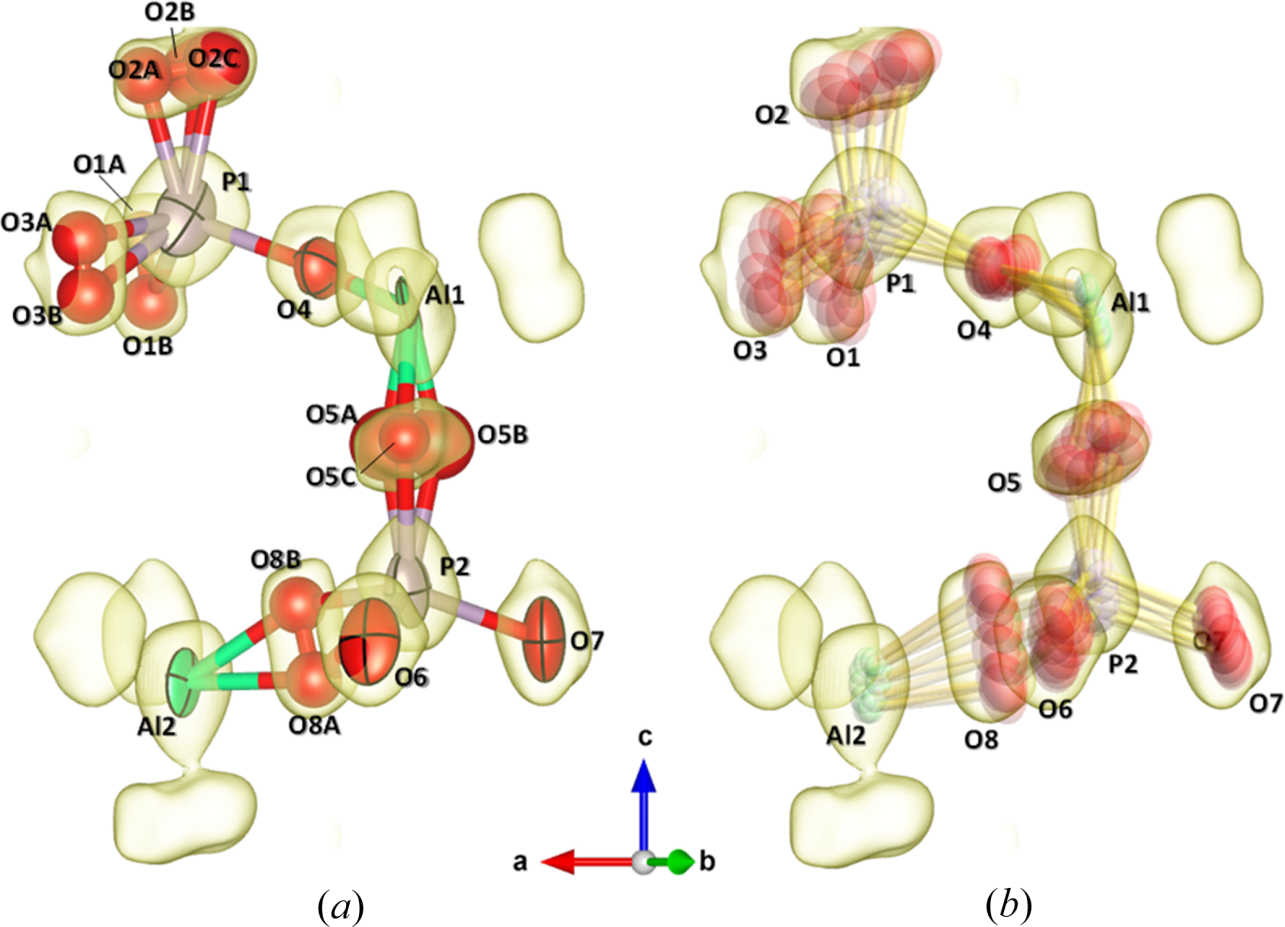
(*a*) 3D averaged and (*b*) (3+1)D crystal structure models with electron density distribution derived from the MEM for asymmetric unit of AlPO_4_-5 structure. Shaded surfaces depict averaged electron densities with isosurface level of 2.0 e Å^−3^ derived from the MEM using the main reflections. In (*b*), the (3+1)D structure models drawn while changing *t* (phase of modulation) in steps of 0.1 from 0 to 0.9 are superposed.

An initial crystal structure based on space group *P*6 was obtained by direct methods, and the resulting model is isotopological to the previously proposed structure with space group *P*6*cc* (Klap *et al.*, 2000 ▸). Structure refinement based on this initial model, with anisotropic atomic displacement parameters (ADPs), resulted in extremely flattened, elongated, or even degenerate displacement ellipsoids for several O atoms. To account for this behaviour, we adopted a split-site model for these O atoms, assigning half or one-third occupancy depending on the number of split positions, with isotropic ADPs, as shown in Fig. 5 ▸. The final *R* factors remained considerably larger (*R*_1_ = 0.0727) compared with the small *R*_int_ value of 0.0151 (see Table 1 ▸).

One possible reason for this discrepancy is that the complex distribution of atomic displacements in the (3+1)D superspace cannot be adequately modelled within split-site structural models. Supporting this interpretation, analysis using MEM, which allows greater flexibility in the electron density distribution than conventional structural models, yielded an excellent agreement factor of *R_F_* = 0.0243.

### 3.3. Analysis of incommensurate structure

The obtained single-crystal diffraction images for dried AlPO_4_-5 exhibit clear satellite peaks with the modulation vector of **q** = 0.36710 (5)c* up to second-order indices of *m*, which were first observed in this study (Fig. 4 ▸). As shown in Fig. 4 ▸, the satellite reflections along the c* direction are absent, indicating that, for the 00*lm* reflections, those with *m* = 1 and 2 are systematically extinct. Based on space group *P*6, the possible (3+1)D superspace groups are therefore limited to two candidates: *P*6(00γ)*h* (No. 168.2; reflection condition: 00*lm*: *m* = 6*n*) and *P*6(00γ)*t* (No. 168.3; 00*lm*: *m* = 3*n*), according to *International Tables for Crystallography*, Vol. C, Table 9.8.3.5. Modulated structure models were then derived using the charge-flipping method based on these two superspace groups. As a result, a consistent modulated structure model was obtained only for *P*6(00γ)*h*. The modulation should be derived from the positional displacements of the host framework, not from occupancy modulation nor displacements of guest molecules, considering the results of the heating experiments under vacuum. Thus, the positional displacement parameters for the first-order harmonics were obtained using *Superflip* (Palatinus & Chapuis, 2007 ▸) and refined up to second-order harmonics using *JANA2020* (Petříček *et al.*, 2023 ▸).

Although the apparent Laue class is 6/*mmm*, whereas space group *P*6 belongs to Laue class 6/*m*, the possibility of merohedral twinning should be considered. However, structure refinements taking into account the possible twin operations (−1, .*m*., .2.), as listed in Table 1.3.4.1 of *International Tables for Crystallography*, Vol. C (Prince, 2004 ▸), did not result in any significant improvement compared with refinements without considering twinning. Furthermore, the final *R* factor is sufficiently low (Table 1 ▸), the anisotropic ADPs yielded physically reasonable ellipsoids, and the Al–O and P–O bond lengths fall within acceptable ranges without the application of any special restraints [Fig. 5 ▸(*b*)]. These observations suggest that the probability of crystal twinning is low. On the other hand, it should be noted that determination of the absolute structure is difficult in this study, because Mo *Kα* radiation was used, and the sample does not contain any heavy elements.

### 3.4. Origin of the modulation

The incommensurate structure observed in AlPO_4_-5 is characterized by rotations of the AlO_4_ and PO_4_ tetrahedra (Figs. 5 ▸ and 6 ▸). As shown in Fig. 5 ▸, the positions of Al and P atoms exhibit minimal displacement with respect to changes in the modulation phase, *t*, whereas the O atoms either trace circular paths around their average positions or oscillate along linear trajectories. This distribution of atomic positions is in good agreement with the averaged electron density distribution derived from the MEM analysis of the main reflections [Fig. 5 ▸(*b*)].

**Figure 6:**
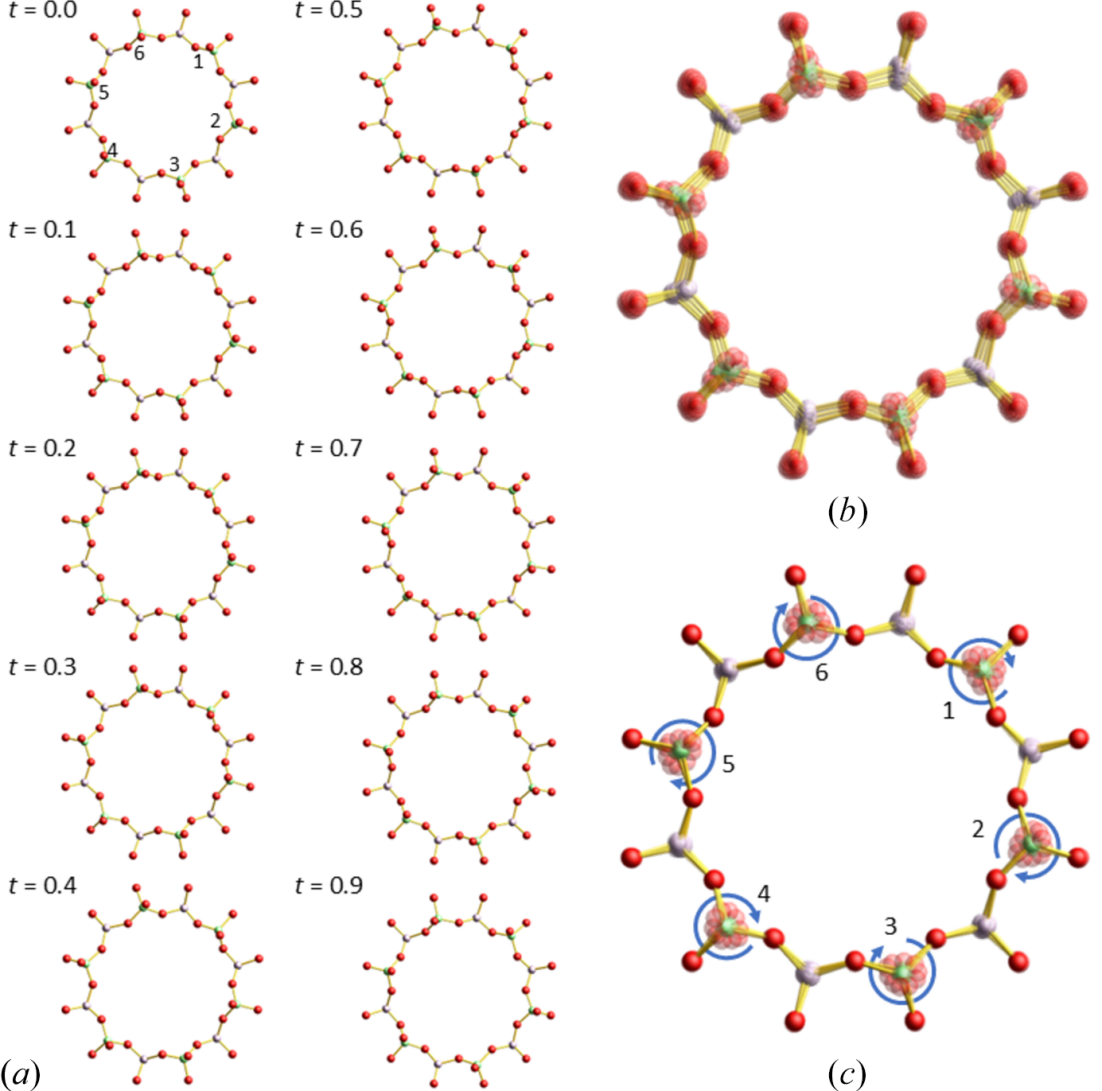
(*a*) The ball-and-stick structure models of the 12-membered ring in the AlPO_4_-5 drawn while changing *t* (phase of modulation) in steps of 0.1 from 0 to 0.9. (*b*) Simply superposed image of the ten images in (*a*). (*c*) Superposed image of the ten images in (*a*), translated so that the basal O atoms of each AlO_4_ or PO_4_ tetrahedron overlap. The numbers 1–6 in (*a*) and (*c*) are assigned for convenience to the apical O atoms of the AlO_4_ tetrahedra. See also video 1 in supporting information.

Let us examine the structural changes with varying modulation phase, *t*. Focusing on the 12-membered ring, it is evident that the apical O atoms of the AlO_4_ tetrahedra—*i.e.* the O atoms in the Al–O–P bonds parallel to the *c* axis—rotate counterclockwise with increasing *t* [Fig. 6 ▸(*a*)]. Owing to the symmetry constraints of the superspace group, *P*6(00γ)*h*, the phases of rotation for apical O atoms located on opposite sides of the 12-membered ring are identical; that is, atoms 1–4, 2–5, and 3–6 in Fig. 6 ▸(*a*) rotate in phase, while adjacent apical O atoms exhibit phase shifts of 2π/3 with respect to each other. Such displacements are also suggested by rigid-unit-mode (RUM) calculations for SiO_4_ tetrahedra in SSZ-24, a silicate zeolite with an isotypic framework of AlPO_4_-5 (Liu *et al.*, 2002 ▸). According to the RUM analysis, the B_1_ mode possesses a threefold rotational softening at **k** = (0, 0, 0.392). The softening of these phonon modes has been proposed to account for the satellite reflections observed at **q** = 0.37c* (Lotti *et al.*, 2016 ▸) and **q** = 0.36710 (5)c* in this study.

When the structural models at different *t* values are simply superposed, the basal O atoms located in the *ab* plane also appear to move [Fig. 6 ▸(*b*)]. However, when the structures are superposed such that these basal O atoms coincide, they overlap almost perfectly [Fig. 6 ▸(*c*)]. This indicates that the basal O atoms within the 12-membered ring translate without changing their relative positions in the *ab* plane. This behaviour can be more intuitively appreciated in supplementary video 1.

On the other hand, when the modulated structure is viewed along a direction perpendicular to the *c* axis, the basal O atoms are seen to undergo undulating displacements along the direction parallel to the *c* axis as *t* varies, in concert with neighbouring O atoms (supporting video 2). During this modulation, the bond lengths and bond angles within the AlO_4_ and PO_4_ tetrahedra remain nearly unchanged—even though no geometrical restraints were imposed in the refinement—indicating that the tetrahedra behave essentially as rigid units. These characteristics of the structural variation with varying *t* apparently resemble the vibrational modes of phonons, as proposed for the origin of diffuse scattering in AlPO_4_-5 (Fischer *et al.*, 2026 ▸).

It should be noted that the aforementioned rotation or vibration represents spatial phase shifts with respect to the modulation phase *t* and does not necessarily imply real dynamic temporal motion. However, in the case of AlPO_4_-5, dynamic motion may indeed play a role. The results from quasi-elastic neutron scattering and molecular dynamics simulations (Cortie *et al.*, 2017 ▸) indicate that, at temperatures above at least 100 K, the O atoms bridging Al and P atoms exhibit motion along circular trajectories. In addition, diffuse scattering measurements at ambient conditions have been interpreted in terms of phonon-like correlated motions within the framework (Fischer *et al.*, 2026 ▸), suggesting that such dynamics can give rise to long-range correlations that manifest as satellite reflections when stabilized as a long-period modulation. In the present study, high-temperature X-ray diffraction experiments reveal that the modulated structure disappears above 335 (5) K, whereas quasi-elastic neutron scattering (QENS) data indicate that O atoms are already mobile at 300 K. If this interpretation of the QENS data is correct, the tetrahedra must be dynamically vibrating while maintaining long-range phase correlations. The persistence of a long-period incommensurate structure in the presence of dynamically moving units, despite thermal vibrational perturbations, is an astonishing phenomenon. Furthermore, as shown by the appearance of satellite reflections upon dehydration in this study, it is reasonable to infer that such dynamic long-period structures are extremely sensitive to subtle changes in external conditions, such as the presence or absence of guest molecules. These findings highlight the intricate interplay between framework flexibility, modulation phenomena, and host–guest interactions in AlPO_4_-5.

## Supplementary Material

Crystal structure: contains datablock(s) global, I. DOI: 10.1107/S205252062600449X/dk5143sup1.cif

Structure factors: contains datablock(s) I. DOI: 10.1107/S205252062600449X/dk5143Isup2.hkl

Video 1. DOI: 10.1107/S205252062600449X/dk5143sup3.mp4

Video 2. DOI: 10.1107/S205252062600449X/dk5143sup4.mp4

Figs S1 and S2. DOI: 10.1107/S205252062600449X/dk5143sup5.pdf

JorIihFWgvi

CCDC reference: 2550293

## Figures and Tables

**Table 1:** Experimental details

	3D Averaged structure	(3+1)D Modulated structure
Crystal data		
Chemical formula	AlO_4_P	AlO_4_P
*M*_r_	122	122
Crystal system, space group	Hexagonal, *P*6	Hexagonal, *P*6(00γ)*h*†
Temperature (K)	295	295
Modulation wavevectors	–	**q** = 0.36710 (5)c*
*a*, *c* (Å)	13.76570 (17), 8.37040 (12)	13.7657 (17), 8.37040 (12)
*V* (Å^3^)	1373.64 (3)	1373.64 (3)
Z	12	12
Radiation type	Mo *K*α	Mo *K*α
μ (mm^−1^)	0.67	0.67
Crystal size (mm)	0.1 × 0.07 × 0.07	0.1 × 0.07 × 0.07

Data collection		
Diffractometer	Rigaku, Synergy Custom, HyPix	Rigaku, Synergy Custom, HyPix
Absorption correction	Empirical	Empirical
No. of measured, independent and observed [*I* > 2σ(*I*)] reflections	24151, 1617, 1614	103791, 8733, 8597
No. of parameters	111	180
*R*_int_	0.015	0.057
(sin θ/λ)_max_ (Å^−1^)	0.624	0.625
*h*,*k*,*l* range	−17 → 17, −17 → 17, −10 → 10	−17 → 17, −17 → 17, −10 → 10, −2 → 2

Refinement		
Refinement method	*F* ^2^	*F* ^2^
No. of reflections	1617	8733
No. of parameters	111	252
*R*[*F*^2^ > 2σ(*F*^2^)], *wR*(*F*^2^), *S* for overall reflections	0.073, 0.138, 1.15	0.082, 0.220, 7.54
*R*[*F*^2^ > 2σ(*F*^2^)], *wR*(*F*^2^), No. of main reflections	–	0.057, 0.184, 1733
*R*[*F*^2^ > 2σ(*F*^2^)], *wR*(*F*^2^), No. of first-order satellite reflections	–	0.066, 0.181, 3531
*R*[*F*^2^ > 2σ(*F*^2^)], *wR*(*F*^2^), No. of second-order satellite reflections	–	0.163, 0.312, 3469
Δρ_max_, Δρ_min_ (e Å^−3^)	0.76, −0.79	0.66, −0.67

Symmetry operations: (1) *x*_1_, *x*_2_, *x*_3_, *x*_4_; (2) −*x*_2_, *x*_1_ − *x*_2_, *x*_3_, *x*_4_ + ⅓; (3) −*x*_1_ + *x*_2_, −*x*_1_, *x*_3_, *x*_4_ + 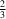; (4) −*x*_1_, −*x*_2_, *x*_3_, *x*_4_ + ½; (5) *x*_2_, −*x*_1_ + *x*_2_, *x*_3_, *x*_4_ + 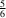; (6) *x*_1_−*x*_2_, *x*_1_, *x*_3_, *x*_4_ + 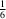.
