# Supplementary material for: Incommensurate structure of AlPO4-5 and its stability
Source: Acta Crystallogr B Struct Sci Cryst Eng Mater. 2026 May 26;82(Pt 3):344–51. doi: 10.1107/S205252062600449X (PMC13238488; doi:10.1107/S205252062600449X)
Supplement: Supplementary file 5 [file b-82-00344-sup5.pdf]

STRUCTURAL SCIENCE
CRYSTAL ENGINEERING
MATERIALS

Volume 82 (2026)

Supporting information for article:

Incommensurate structure of AlPO4-5 and its stability

Kazuki Komatsu, Takuji Ikeda and Tetsuya Kodaira

Site	$x_1 - x_4$	$x_2 - x_4$	$x_3 - x_4$
P1	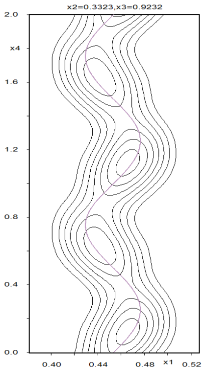	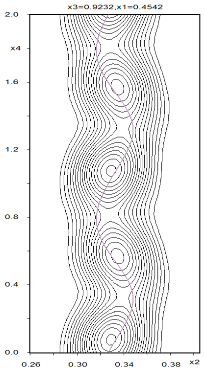	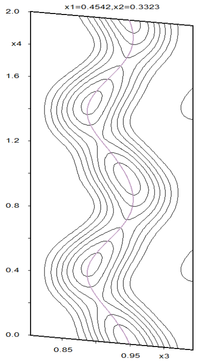
P2	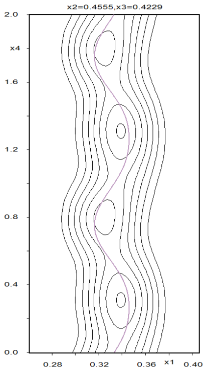	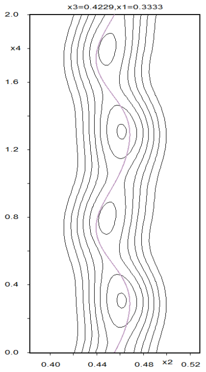	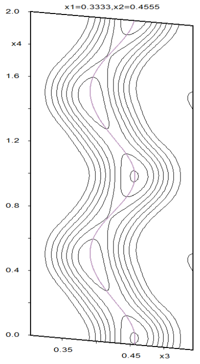
Al1	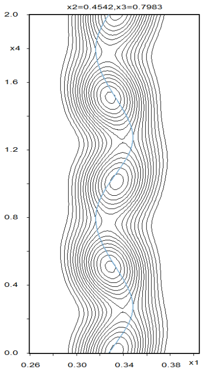	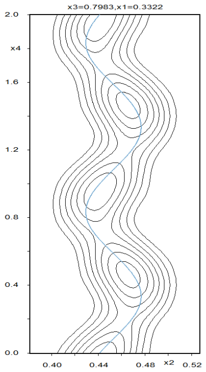	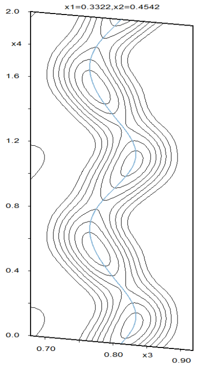
Al2	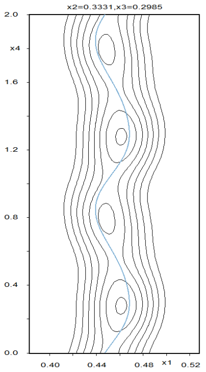	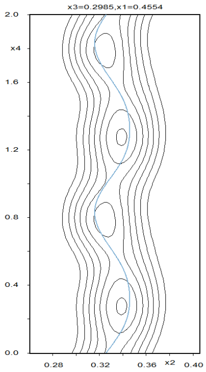	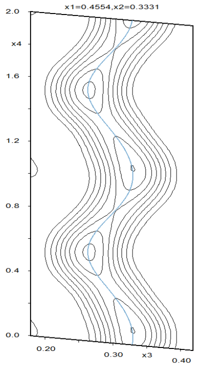

Supplementary Figure 1

Contour maps of the Fourier electron density for (x_1, x_4), (x_2, x_4) and (x_3, x_4) cross-sections through respective sites.

Site	$x_1 - x_4$	$x_2 - x_4$	$x_3 - x_4$
O1	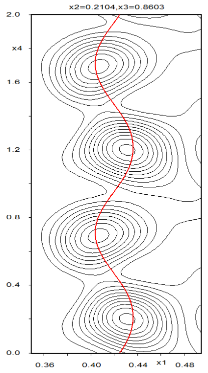	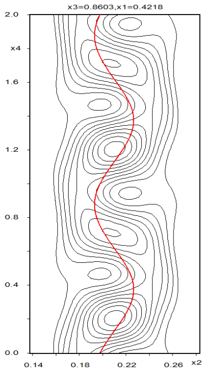	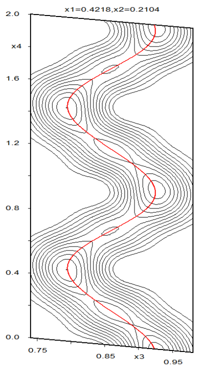
O2	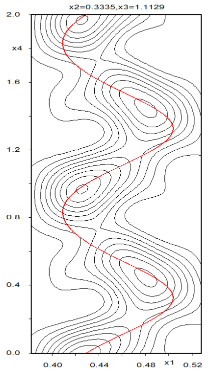	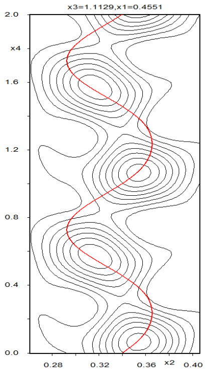	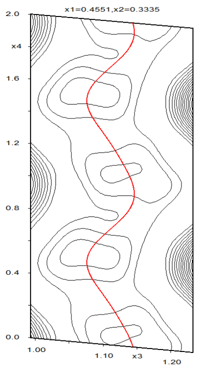
O3	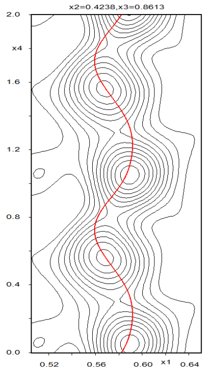	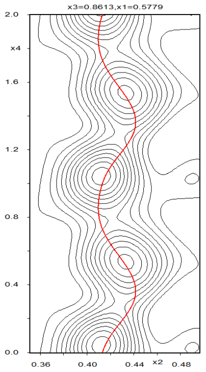	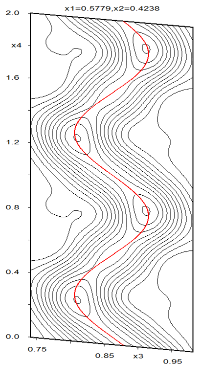
O4	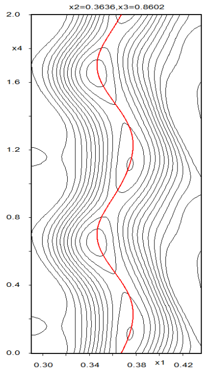	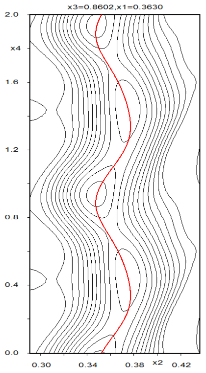	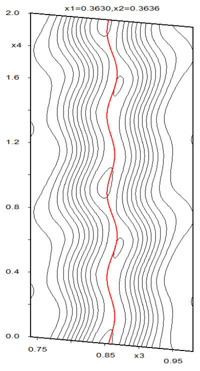

Supplementary Figure 1 (cont.)

Site	$x_1 - x_4$	$x_2 - x_4$	$x_3 - x_4$
O5	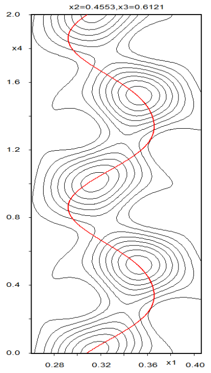	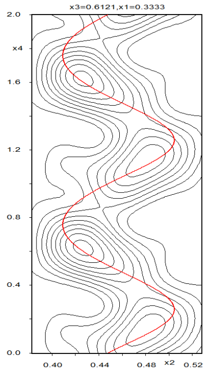	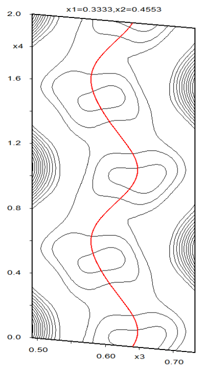
O6	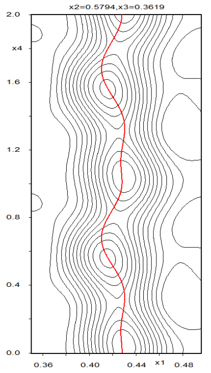	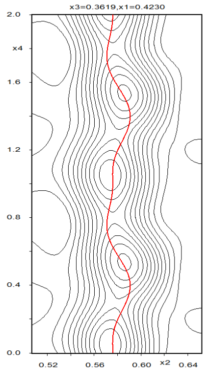	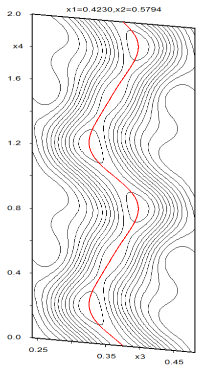
O7	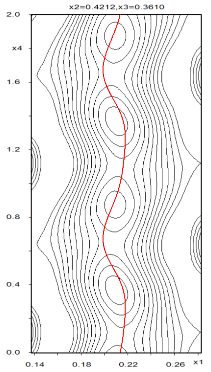	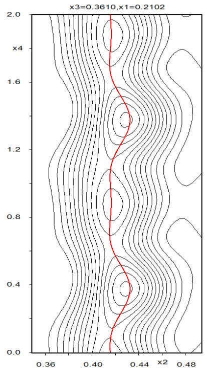	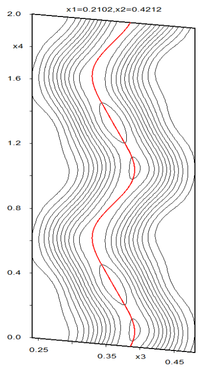
O8	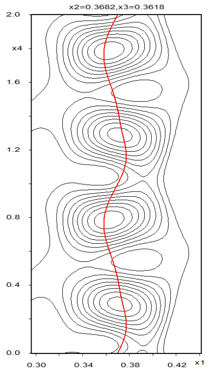	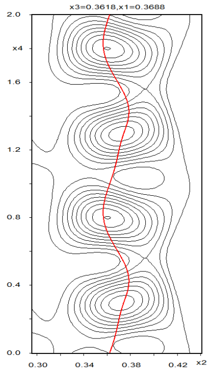	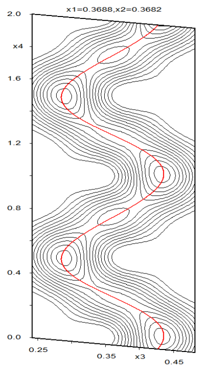

Supplementary Figure 1 (cont.)

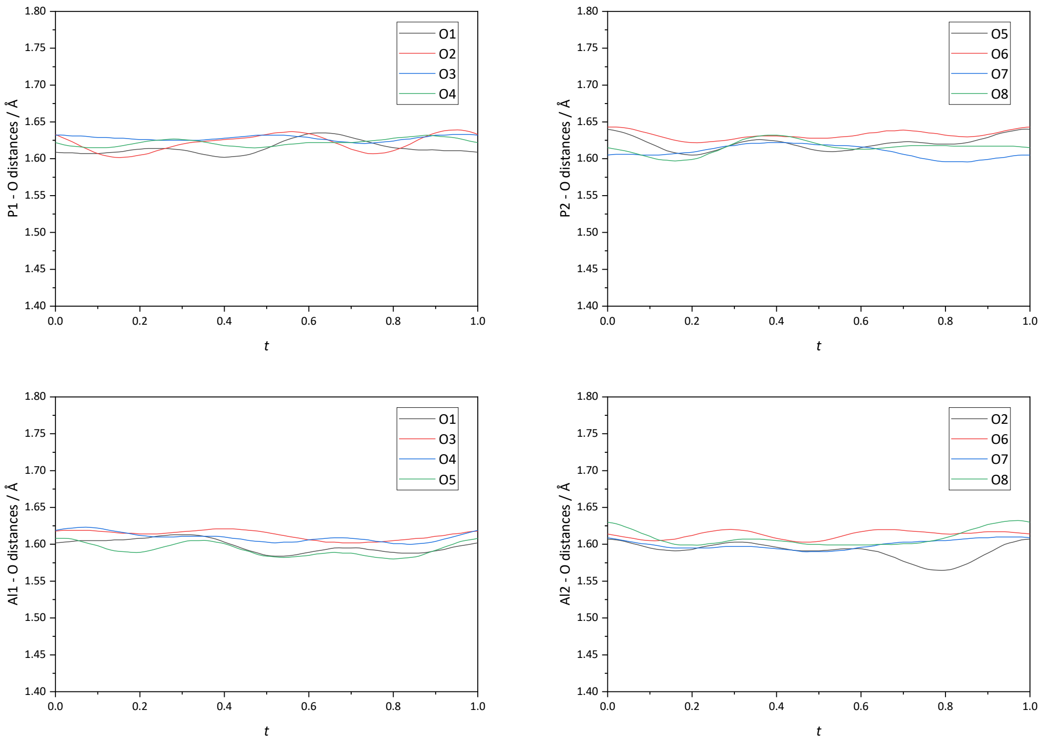

Supplementary Figure 2

P-O and Al-O bond distances as a function of modulation phase, *t*.
